# Mps1 dimerization and multisite interactions with Ndc80 complex enable responsive spindle assembly checkpoint signaling

**DOI:** 10.1093/jmcb/mjaa006

**Published:** 2020-03-26

**Authors:** Ping Gui, Divine M Sedzro, Xiao Yuan, Sikai Liu, Mohan Hei, Wei Tian, Najdat Zohbi, Fangwei Wang, Yihan Yao, Felix O Aikhionbare, Xinjiao Gao, Dongmei Wang, Xuebiao Yao, Zhen Dou

**Affiliations:** 1 MOE Key Laboratory of Membraneless Organelle and Cellular Dynamics, Hefei National Laboratory for Physical Sciences at the Microscale, School of Life Sciences, University of Science and Technology of China, Hefei 230027, China; 2 Keck Center for Cellular Dynamics and Organoids Plasticity, Morehouse School of Medicine, Atlanta, GA 30310, USA; 3 Key Laboratory of Molecular Biophysics of the Ministry of Education, College of Life Science and Technology, Huazhong University of Science and Technology, Wuhan 430074, China; 4 Life Sciences Institute and Innovation Center for Cell Signaling Network, Zhejiang University, Hangzhou 310058, China

**Keywords:** mitosis, spindle assembly checkpoint, kinetochore, Mps1 kinase, Ndc80 complex

## Abstract

Error-free mitosis depends on accurate chromosome attachment to spindle microtubules, which is monitored by the spindle assembly checkpoint (SAC) signaling. As an upstream factor of SAC, the precise and dynamic kinetochore localization of Mps1 kinase is critical for initiating and silencing SAC signaling. However, the underlying molecular mechanism remains elusive. Here, we demonstrated that the multisite interactions between Mps1 and Ndc80 complex (Ndc80C) govern Mps1 kinetochore targeting. Importantly, we identified direct interaction between Mps1 tetratricopeptide repeat domain and Ndc80C. We further identified that Mps1 C-terminal fragment, which contains the protein kinase domain and C-tail, enhances Mps1 kinetochore localization. Mechanistically, Mps1 C-terminal fragment mediates its dimerization. Perturbation of C-tail attenuates the kinetochore targeting and activity of Mps1, leading to aberrant mitosis due to compromised SAC function. Taken together, our study highlights the importance of Mps1 dimerization and multisite interactions with Ndc80C in enabling responsive SAC signaling.

## Introduction

Faithful distribution of the duplicated genome into two daughter cells during mitosis depends on proper kinetochore–microtubule attachments. Defects in kinetochore–microtubule attachments result in chromosome mis-segregation, causing aneuploidy, a hallmark of cancer (Santaguida and Amon, 2015). To ensure accurate chromosome segregation, a cellular signaling pathway called spindle assembly checkpoint (SAC) monitors kinetochore bi-orientation and controls the metaphase to anaphase transition. Until all kinetochores are attached to microtubules properly, cells enter anaphase (London and Biggins, 2014; Huang et al., 2019). Biochemically, the anaphase promoting complex/cyclosome (APC/C, an ubiquitin E3 ligase) activity triggers metaphase–anaphase transition by promoting ubiquitin-mediated proteolysis of two key mitotic factors, cyclin B and securin (Watson et al., 2019). SAC signaling catalyzes the generation of the mitotic checkpoint complex (MCC), which directly inhibits the activity of APC/C to hold the cell at metaphase (London and Biggins, 2014; Musacchio, 2015).

The SAC consists of a number of core signaling molecules including Mad1, Mad2, Mad3/BubR1, Bub1, Bub3, and Mps1. Besides these factors, Cdk1–cyclin B and Aurora B play complicated but indispensable roles in SAC functional integrity (Santaguida et al., 2011; Hayward et al., 2019). Among the SAC components, *monopolar spindle 1* (*Mps1*) was originally identified in budding yeast as a gene required for spindle pole body duplication. This kinase is evolutionarily conserved as its orthologues in different species from fungi to mammals have been identified and shown to play an essential role in the SAC (Pachis and Kops, 2018). It is well established that Mps1 is an upstream component of SAC, as it is required to recruit Mad1 and Mad2 to the kinetochores with improper microtubule attachment (Martin-Lluesma et al., 2002; Pachis and Kops, 2018; Dou et al., 2019). Through phosphorylating the multiple Met-Glu-Leu-Thr (MELT) motifs of Knl1, Mps1 promotes SAC signaling by enhancing Knl1-mediated recruitment of Bub1 and Bub3 to the kinetochores (Musacchio, 2015). Hierarchically, Bub1 thereafter recruits BubR1/Bub3 to the kinetochore through hetero-dimerization with BubR1 (Overlack et al., 2015; Zhang et al., 2015). Subsequently, Mps1 phosphorylates Bub1 and Mad1. These phosphorylation events enable the recruitment of Mad1/Mad2 to the unattached kinetochores, which is mediated in two parallel pathways, Knl1–Bub3–Bub1 and Rod–ZW10–Zwilch (Dou et al., 2019). They also facilitate Mad2–Cdc20 binding and hence the formation of the MCC (Luo et al., 2018). Evidence suggests that Mps1 also participates in regulating chromosome alignment (Maure et al., 2007; Jelluma et al., 2008; Maciejowski et al., 2017). However, it is still debatable whether Mps1 plays a major role in facilitating chromosome biorientation in human cells (Dou et al., 2015). Besides its role in SAC and chromosome alignment, it has been demonstrated that Mps1 kinase activity is crucial for the kinetochore expansion in early prometaphase (Suzuki and Varma, 2018).

How kinetochore localization of Mps1 is dynamically regulated has only begun to be appreciated. It is clear that Aurora B kinase activity and the outer-layer kinetochore Ndc80 complex (Ndc80C) are required for Mps1 localization to kinetochores as evident from our recent work and others (Santaguida et al., 2010; Dou et al., 2011; Saurin et al., 2011; Heinrich et al., 2012; Nijenhuis et al., 2013; Zhu et al., 2013). Besides Aurora B, Cdk1 regulates Mps1 kinetochore localization and activity positively (Morin et al., 2012; Alfonso-Perez et al., 2019; Hayward et al., 2019). On the contrary, Mps1 itself negatively regulates its kinetochore localization (Hewitt et al., 2010; Jelluma et al., 2010; Santaguida et al., 2010; Thebault et al., 2012; Wang et al., 2014). Recently, several studies demonstrated that Mps1 competes with microtubules to bind to Ndc80C and proposed that competition between Mps1 and microtubules for Ndc80C binding serves as a direct mechanism for sensing unattached kinetochores (Hiruma et al., 2015; Ji et al., 2015). Despite these progresses, our understanding of the Mps1 kinetochore recruitment and release is still insufficient. In this study, we systematically examined the role of each Mps1 module in mediating Mps1 kinetochore localization. Our study provides shreds of evidence that multisite interactions between Mps1 and Ndc80C enable effective kinetochore localization of Mps1. Furthermore, we demonstrated that the Mps1 C-terminal fragment-mediated dimerization contributes to maximal kinetochore localization and that the Mps1 C-tail is essential for the functional integrity of SAC signaling.

## Results

### Multisite interactions between Mps1 and Ndc80C mediate Mps1 localization

**Figure 1:**
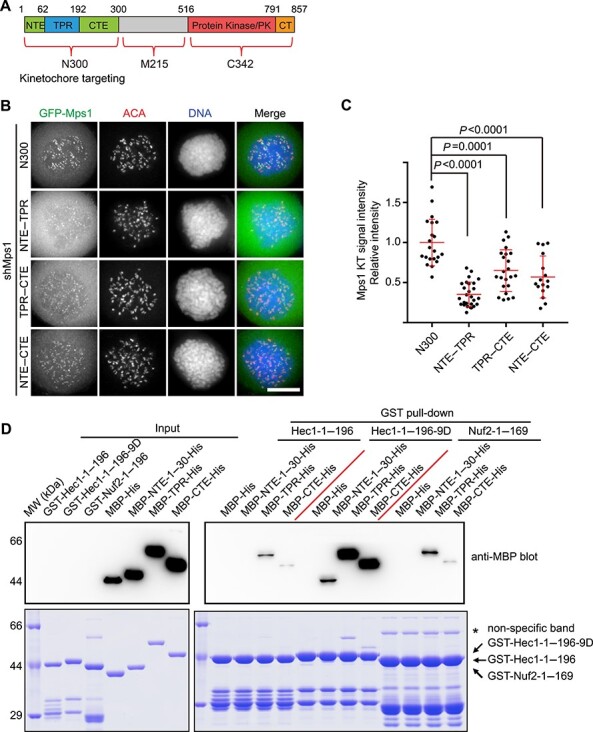
The multisite interactions between Mps1 and Ndc80C mediate Mps1 kinetochore localization. (**A**) Schematic representation of human Mps1 protein domain organization and designated fragments. (**B**) Representative immunofluorescence images of HeLa cells transfected with shMps1 and different shMps1-resistant GFP-Mps1 constructs as indicated. After 36 h of transfection, cells were treated with nocodazole plus MG132 for 2 h. Cells were then fixed and co-stained for ACA (red) and DNA (blue). Scale bar, 10 μm. (**C**) Bar graph illustrating kinetochore (KT) intensity of different GFP-Mps1 proteins treated as in **B**. Bars represent the mean kinetochore intensity (±SD) normalized to the values of GFP-Mps1^N300^ group. Each dot represents one cell (>30 cells from three independent experiments). Student’s *t*-test was used to calculate *P*-values. (**D**) GST-Hec1^1–196^, GST-Hec1^1–196-9D^, or GST-Nuf2^1–169^-bound agarose beads were used as affinity matrices to absorb different MBP-tagged Mps1truncation proteins purified from *Escherichia coli*. Pull-downs were analyzed by SDS–PAGE and probed by anti-MBP blotting. Arrow indicates specific binding protein bands. Asterisk indicates the non-specific bands.

The cellular localization of mitotic kinases must be governed accurately, so that they can precisely phosphorylate their substrates (Ubersax and Ferrell, 2007). It is well established that the Mps1 N-terminal fragment (amino acids 1–300, designated as Mps1^N300^) is required and sufficient for its kinetochore localization (Liu et al., 2003). Mps1^N300^ comprises three modules: the N-terminal extension (NTE, amino acids 1–61), the tetratricopeptide repeat domain (TPR, amino acids 62–192), and the C-terminal extension (CTE, amino acids 193–300, see Figure 1A for the schematic representation). To define the contribution of each module in the kinetochore localization of Mps1, we aim to examine the localization of GFP-NTE, GFP-TPR, and GFP-CTE, respectively. To rule out the interference of endogenous Mps1, we applied a protocol of co-transfection of Mps1 shRNA (shMps1) together with different shMps1-resistant plasmids at a ratio of 3:1. As shown by Supplementary Figure S1A and B, Mps1 signal is almost invisible in shMps1-transfected cells, indicating the potent efficacy of shMps1. We also note that the shMps1 had been previously verified (Jelluma et al., 2008; Dou et al., 2015). Mps1^N300^ displayed clear kinetochore signal, as judged by the colocalization with centromere (ACA staining). However, no kinetochore signal was observed in cells expressing GFP-NTE, GFP-TPR, or GFP-CTE (Supplementary Figure S1C and D). Next, we examined the localization of different Mps1 truncations that cover two modules. In this situation, all Mps1 truncation proteins (Mps1^NTE–TPR^, Mps1^TPR–CTE^, and Mps1^NTE–CTE^) displayed weak, but clear, kinetochore localization (Figure 1B and C). Compared with Mps1^N300^, the kinetochore signal intensities of these different truncations are significantly weaker, suggesting that each module contributes to Mps1 kinetochore localization. We note that the protein expression levels of different truncations are in a comparable level, ruling out the possibility that the difference of kinetochore localization intensity is due to the protein expression variation (Supplementary Figure S1E).

The NTE plays an important role in mediating Mps1 kinetochore localization by direct interaction with the highly expressed in cancer 1 (Hec1) calponin-homology (CH) domain (Nijenhuis et al., 2013; Dou et al., 2015). Our secondary structure analysis (PSIPRED software) indicated the presence of a long α-helix (amino acids 13–27, NTE^helix-1^) and a second short α-helix (amino acids 50–58, NTE^helix-2^) within the NTE. In addition, the first long α-helix is highly conserved among different species (Supplementary Figure S2A). To test the importance of these two α-helices, we constructed plasmids expressing GFP-tagged Mps1 protein without NTE^helix-1^ and NTE^helix-2^, respectively. Compared with Mps1^WT^, the kinetochore signal of Mps1^ΔN30^ was nearly invisible, suggesting the key role of the fragment of amino acids 1–30 in mediating Mps1 kinetochore targeting. Intriguingly, the kinetochore localization of Mps1^Δ31–60^ was stronger than wild-type Mps1 (Mps1^WT^) (Supplementary Figure S2B). This may imply that Mps1^Δ31–60^ has reduced kinase activity, consistent with the recent report that Mps1 fragment of residues 40–49 has an inhibitory effect on its kinase activity (Combes et al., 2018). We further examined the localization of these truncations in the background of kinase dead Mps1 (Mps1^KD^). As shown in Supplementary Figure S2C and D, Mps1^ΔN30-KD^ has significantly decreased kinetochore localization, whereas the localization of Mps1^Δ31–60-KD^ is as strong as Mps1^KD^. We note that the expression levels of these truncations are similar (Supplementary Figure S2E). Taken together, we concluded that NTE^helix-1^ plays a key role in mediating Mps1 kinetochore localization. Although NTE^31–60^ is dispensable for the kinetochore localization of Mps1, it may have another role in functional integrity of Mps1 activity. During the course of this study, the Kops group confirmed our conclusion that the first α-helix fragment of Mps1 is critical for its kinetochore localization (Pachis et al., 2019).

The kinetochore signal intensities of different Mps1 truncations indicate the different binding affinities between these truncations and Ndc80C. To provide direct biochemical evidence, we generated plasmids expressing different MBP-tagged Mps1 truncations. GST-tagged Ndc80C^Bonsai^ was expressed and used as an affinity matrix to pull down different Mps1 truncations. As shown in Supplementary Figure S2F, Mps1^N300^ and Mps1^TPR–CTE^ have a strong binding affinity with Ndc80C^Bonsai^. Compared with Mps1^N300^, Mps1^TPR^ and Mps1^NTE–TPR^ display a weaker binding affinity with Ndc80C^Bonsai^. Consistent with a previous study (Hiruma et al., 2015), these data indicate that TPR mediates Mps1–Ndc80C binding directly, and the combination of TPR with NTE/CTE enhances the binding affinity. Taken together, we envision that the kinetochore localization of Mps1 is mediated by multisite interactions between Mps1 and Ndc80C.

It is well documented that the microtubule binding activity of Ndc80C is controlled by Aurora B phosphorylation of the Hec1 N-terminal 80-amino acid unstructured region (termed N-tail hereafter) (Cheeseman et al., 2006; DeLuca et al., 2006; Ciferri et al., 2008). Concurrently, Aurora B phosphorylation toward the Hec1 N-tail enhances the Mps1–Ndc80C binding significantly (Nijenhuis et al., 2013; Zhu et al., 2013; Ji et al., 2015). To address the detailed biochemical mechanism, GST-tagged Hec1^1–196^, Hec1^1–196-9D^ (mimicking phosphorylation by Aurora B), and Nuf2^1–169^ were used as the affinity matrix to pull down MBP-tagged Mps1^NTE-1–30^ (Mps1^NTE-1–30^ was used due to better protein expression), Mps1^TPR^, Mps1^CTE^, respectively. Compared with Hec1^1–196^, Nuf2^1–169^ has a comparable binding affinity toward Mps1^TPR^ and Mps1^CTE^ (Figure 1D). This suggests that both Hec1 and nuclear filament-containing protein 2 (Nuf2) CH domains contribute to binding with Mps1^TPR^ and Mps1^CTE^. Previously, studies conducted by our group and Yu group indicated that the Hec1 N-tail has an auto-inhibitory effect on both Hec1 itself and Nuf2 (Ji et al., 2015; Zhao et al., 2019). Consistent with these observations, we found that Hec1^1–196-9D^ has a remarkably higher affinity with all three Mps1 modules (Figure 1D). Thus, we concluded that the Hec1 N-tail interferes with both the Hec1 and Nuf2 CH domains until this inhibition was relieved by Aurora B phosphorylation.

### The C-terminal fragment of Mps1 promotes its kinetochore localization

**Figure 2:**
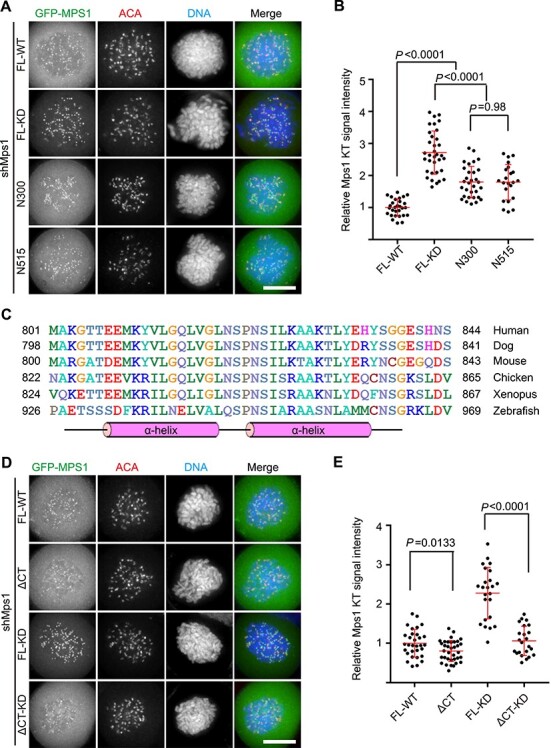
Mps1 C-terminal fragment promotes its kinetochore localization. (**A** and **D**) Representative immunofluorescence images of HeLa cells transfected with shMps1 and different shMps1-resistant GFP-Mps1 constructs as indicated. After 36 h of transfection, cells were treated with nocodazole plus MG132 for 1 h. Then cells were fixed and co-stained for ACA (red) and DNA (blue). Scale bar, 10 μm. (**B** and **E**) Bar graphs illustrating kinetochore intensity of different GFP-Mps1 fusion proteins treated as in **A** and **D**. Bars represent the mean kinetochore intensity (±SD) normalized to the values of Mps1^WT^. Each dot represents one cell (>30 cells from three independent experiments). Student’s *t*-test was used to calculate *P*-values. (**C**) Multiple sequence alignment of Mps1 proteins from different species as indicated. The sequence alignment was done by ClustalW2 software. The secondary structure was predicted using PSIPRED online tool (http://bioinf.cs.ucl.ac.uk/psipred/).

To further investigate the mechanism of Mps1 kinetochore recruitment, we first compared the kinetochore signal intensity of Mps1^WT^, Mps1^KD^, and Mps1^N300^. Consistent with previous studies (Hewitt et al., 2010; Santaguida et al., 2010), the kinetochore signal of Mps1^KD^ is significantly stronger than Mps1^WT^, suggesting that the kinase activity of Mps1 somehow negatively regulates its localization (Figure 2A and B). Interestingly, the kinetochore signal of Mps1^KD^ was clearly stronger than Mps1^N300^. It suggests that Mps1 fragment of amino acids 301–857 (referred to as Mps1^C557^) in its inactive state may enhance the localization of Mps1^N300^. Careful examination of the localization of Mps1^C557^ (both the protein kinase domains in WT and KD versions) rules out the presence of an additional kinetochore binding domain/motif (Supplementary Figure S3A). Comparing the kinetochore localization intensity of Mps1^1–515^ with Mps1^N300^ indicates that the Mps1^M215^ fragment (amino acids 301–515) is not involved in kinetochore localization (Figure 2A). Previously, we found that Mps1^ΔM215-KD^ kinetochore signal was clearly stronger than that of Mps1^ΔM215-WT^ and Mps1^N300^ (Dou et al., 2015). These data suggest that the Mps1^C342^ fragment (amino acids 516–857), which encompasses the protein kinase (PK, amino acids 516–792) domain and the C-tail (CT, amino acids 793–857), enhances Mps1 kinetochore localization.

The function of Mps1^CT^ fragment remains poorly understood. Our bioinformatics analysis showed that Mps1^CT^ contains a highly conserved region and the secondary structure prediction indicated that this conserved region includes two α-helices (Figure 2C). Therefore, we speculated that Mps1^CT^ might have a key function in promoting Mps1 localization. Indeed, the kinetochore localization of Mps1^ΔCT^ is clearly weaker than Mps1. Furthermore, the kinetochore signal intensity of Mps1^ΔCT-KD^ is remarkably weaker than Mps1^KD^ (Figure 2D and E). Note that all the truncations examined were expressed at a comparable level (Supplementary Figure S3B). To determine the contributions of Mps1^PK^ and Mps1^CT^ in boosting Mps1 kinetochore localization, a series of different Mps1 truncation plasmids were constructed as illustrated in Supplementary Figure S3C. Compared with Mps1^N300^, the kinetochore signal of Mps1^N300-CT^ was clearly elevated. Regarding Mps1^N300-PK-KD^, a moderately increased kinetochore signal was observed (Supplementary Figure S3C and D). As all these Mps1 mutants were expressed at a level comparable to GFP-Mps1^N300^ (Supplementary Figure S3E), we concluded that the different kinetochore staining observed was not due to variable protein expression levels. These data suggest that both Mps1^PK-KD^ and Mps1^CT^ contribute to boosting kinetochore localization. Taken together, we concluded that the Mps1^CT^ fragment and Mps1^PK^ domain contribute to the maximal initial kinetochore localization of inactive Mps1.

### The Mps1 C-terminal fragment contributes to Mps1 dimerization

**Figure 3:**
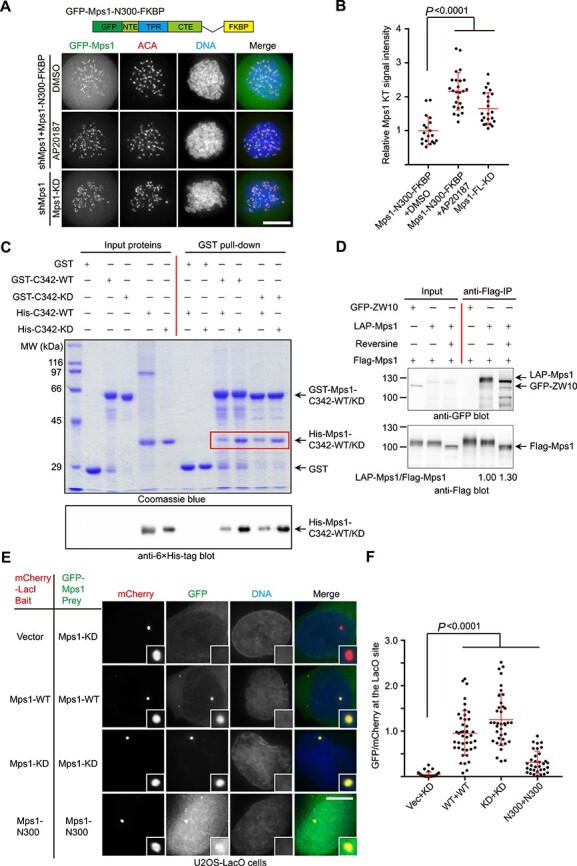
Mps1 C-terminal fragment mediates Mps1 dimerization. (**A**) Representative immunofluorescence images of HeLa cells transfected with shMps1 and shMps1-resistant GFP-Mps1^N300^-FKBP construct as indicated. After 36 h of transfection, cells were treated with nocodazole plus MG132 for 1 h. One group of cells was treated with AP20187 for 30 min. Then cells were fixed and co-stained for ACA (red) and DNA (blue). Scale bar, 10 μm. (**B**) Bar graph illustrating kinetochore intensity of GFP-Mps1^N300^-FKBP fusion protein in cells treated as in **A**. Bars represent the mean kinetochore intensity (±SD) normalized to the values of DMSO group. Each dot represents one cell (>30 cells from three independent experiments). Student’s *t*-test was used to calculate *P*-values. (**C**) GST, GST-Mps1^C342-WT^, or Mps1^C342-KD^-bound agarose beads were used as affinity matrices to absorb purified 6× His-tagged Mps1^C342-WT^ or Mps1^C342-KD^ fusion protein. Pull-downs were analyzed by SDS-PAGE and western blotting using anti-6× His tag antibody. (**D**) 293T cells were co-transfected with Flag-Mps1 together with GFP-ZW10 (negative control) and LAP-Mps1, respectively. After 24 h, one group of cells was treated with reversine for 2 h. The cells were collected and lysed, and immunoprecipitation was carried out using anti-Flag M2 beads. Immunoprecipitation samples were resolved by western blotting using anti-GFP antibody and anti-Flag antibody, respectively. The normalized ratio of LAP-Mps1 signal to Flag-Mps1 signal is shown below in lanes 5 and 6. (**E**) Representative immunofluorescence images of U2OS-LacO cells co-expressing different mCherry-LacI-Mps1 (bait) and GFP-Mps1 (pray) constructs as indicated. After 24 h of transfection, cells were fixed and stained with DAPI. The boxed areas are shown magnified in the right panels. Scale bar, 10 μm. (**F**) Bar graph illustrating intensity of different GFP-Mps1 proteins colocalized with different mCherry-LacI baits as indicated in **E**. Bars represent the mean intensity (±SD) normalized to the values of Mps1-WT plus Mps1-WT (WT+WT) group. Each dot represents one cell (>30 cells from three independent experiments). Student’s *t*-test was used to calculate *P*-values.

Dimerization-induced allostery is an important regulatory mechanism for many protein kinases (Lavoie et al., 2014). Several publications had already provided pieces of evidence to support Mps1 functions as a dimer *in vivo* (Kang et al., 2007; Hewitt et al., 2010; Lee et al., 2012) or proposed such model (Jelluma et al., 2010). In addition, Mps1^C342^ itself does not have the kinetochore localization ability. Therefore, we reasoned that Mps1^C342^ might boost the Mps1 kinetochore localization through forming a dimer. To test our hypothesis, GFP-tagged Mps1^N300^-FKBP fusion protein was expressed exogenously and its localization was examined. Indeed, compared with cells treated with DMSO, treatment of small molecular AP20187 boosted the kinetochore localization of fusion protein robustly (Figure 3A). As a control, induced dimerization of Mps1^C557^ fails to localize to the kinetochore (Supplementary Figure S4A), proving that the enhanced kinetochore localization is caused by dimerized Mps1^N300^. Chemical-induced dimerization of Mps1^N300^-FKBP phenocopies the localization of Mps1^KD^, strongly suggesting that the Mps1^C342-KD^ fragment enhances the kinetochore localization of Mps1 through dimerization. Quantification of the kinetochore signal indicates that artificially dimerized Mps1^N300^-FKBP binds to the kinetochore more strongly than Mps1^KD^ (Figure 3B), indicating that the dimerization affinity of Mps1^KD^ is weaker than chemical-induced dimerization. Next, we generated the construct expressing Mps1 protein fusion with the coiled-coil domain of mitotic centromere-associated kinesin (MCAK), which mediates dimerization (Moore and Wordeman, 2004). Consistent with our expectation, MCAK-mediated dimerization enhances the kinetochore localization of Mps1 fusion protein clearly (Supplementary Figure S4B–D).

To prove that Mps1^C342^ has the ability to form a dimer, we performed biochemical analysis. GST-tagged Mps1^C342^ or Mps1^C342-KD^ was purified and used as a bait to pull down 6× His-tagged Mps1^C342-WT/KD^. As shown in Figure 3C, GST-Mps1^C342-WT/KD^ pulls down 6× His-Mps1^C342-WT/KD^. Interestingly, both GST-Mps1^C342-WT^ and GST-Mps1^C342-KD^ pull down more abundant 6× His-Mps1^C342-KD^. This observation suggests that Mps1^C342-KD^ has a higher inter-molecular binding affinity than Mps1^C342-WT^. Our immuno-precipitation assays also demonstrated that Flag-Mps1 pulls down LAP-Mps1, but not GFP-ZW10, ruling out the non-specific binding between Flag-Mps1 and LAP-Mps1 (Figure 3D). The difference in the amount of LAP-Mps1 pulled down by Flag-Mps1 cells treated with or without reversine is not significant, probably due to other domain of full-length Mps1 (such as N300) that contributes to dimerization. To further verify the contribution of C-tail to Mps1 dimerization, we compared the ability of GST-Mps1^C342-KD^ and GST-Mps1^PK-KD^ to pull down 6× His-Mps1^C342^. Consistent with our prediction, GST-Mps1^C342-KD^ pulls down a significant amount of 6× His-Mps1^C342-KD^ than Mps1^PK-KD^, suggesting that C-tail enhances dimerization of Mps1^C342^ (Supplementary Figure S4E). However, we could not detect the direct binding between two C-tail fragments (Supplementary Figure S4F).

To demonstrate the Mps1–Mps1 self-association, we examined the localization of different Mps1 fusion proteins using a LacI/LacO-based *in vivo* interaction assay. Different mCherry-tagged Mps1 constructs were fused to the Lac repressor (LacI) and expressed as baits in U2OS cells that have a stably integrated LacO array (Janicki et al., 2004). GFP-tagged Mps1 fusion proteins were exogenously expressed as pray to test the Mps1–Mps1 interaction. Tethering Mps1^KD^ to the LacO array resulted in GFP-Mps1^KD^ recruitment. Tethering Mps1^WT^ to the LacO array also resulted in GFP-Mps1^WT^ recruitment, but to a less extent as judged by quantification of the colocalization signal (Figure 3E and F). On the contrary, mCherry-LacI-Mps1^N300^ bait protein can only tether a small proportion of GFP-Mps1^N300^ as there is strong cytoplasmic GFP-Mps1^N300^. We also utilized bimolecular fluorescence complementation (BiFC) assay to pinpoint the Mps1 domain/fragment mediating dimerization as reported (Xia et al., 2014). As a control, no signal was observed in BiFC paired by YFPN-Mps1^N300^ and YFPC-Mps1^C342^ in the co-expressed cells (Supplementary Figure S4G). Weak BiFC signal was observed in cells expressing YFPN-Mps1^N300^ and YFPC-Mps1^N300^, supporting the previous observation that both Mps1^NTE^ and Mps1^TPR^ contribute to dimerization (Thebault et al., 2012). On the contrary, bright YFP signal was observed in YFPN-Mps1^PK^ and YFPC-Mps1^PK^ co-transfected cells, as well as in YFPN-Mps1^C342^ and YFPC-Mps1^C342^ co-transfected cells. The YFP signal was enhanced in the presence of Reversine treatment, suggesting that kinase activity somehow negatively regulates the dimerization mediated by Mps1^C342^ (Supplementary Figure S4G and H). Taken together, these data suggest that Mps1 dimerization is mainly mediated by C342 fragment *in vivo* and kinase activity weakens its dimerization. C-tail is essential for Mps1^C342^-mediated dimerization but insufficient to form a dimer alone.

### The Mps1 C-tail is essential for SAC functional integrity

**Figure 4:**
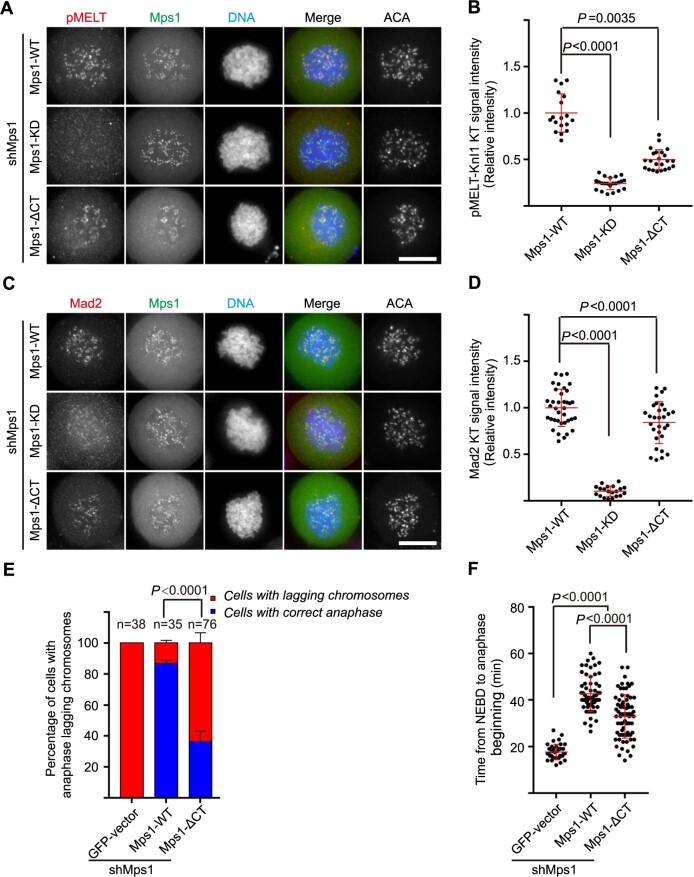
Mps1 C-tail is required for the functional integrity of SAC. (**A** and **C**) Representative immunofluorescence images of HeLa cells transfected with shMps1 and different shMps1-resistant GFP-Mps1 constructs as indicated. After 36 h of transfection, cells were treated with nocodazole plus MG132 for 1 h. Then cells were fixed and co-stained for pMETL-Knl1 (red, **A**) or Mad2 (red, **C**), DNA (blue), and ACA (in gray scale). Scale bar, 10 μm. (**B** and **D**) Bar graphs illustrating kinetochore intensity of pMETL-Knl1 as in **A** and Mad2 as in **C**. Bars represent the mean intensity (±SD) normalized to the values of Mps1^WT^ group. Each dot represents one cell (>18 cells from three independent experiments). Student’s *t*-test was used to calculate *P*-values. (**E**) Bar graph illustrating percentage of cells treated as indicated with anaphase lagging chromosomes. Cells were co-transfected with shMps1, mCherry-H2B, and different GFP-Mps1 plasmids, respectively. Bars indicate mean±SD (>30 cells from three independent experiments). Student’s *t*-test was used to calculate *P*-values. (**F**) Scatter plot of the time from NEBD to anaphase beginning in cells co-transfected with shMps1, different GFP-Mps1, and mCherry-H2B. Bars indicate mean±SD. Each dot represents one cell (>30 cells from three independent experiments). Student’s *t*-test was used to calculate *P*-values.

Having demonstrated the importance of Mps1^CT^ in mediating its kinetochore localization, we further examined whether Mps1^CT^ is critical for SAC function. For this purpose, HeLa cells were co-transfected with shMps1 together with GFP-tagged Mps1^WT^, Mps1^KD^, and Mps1^ΔCT^, respectively. After 36 h, cells were fixed and stained for pMELT-Knl1 and Mad2, respectively. We observed clear pMELT-Knl1 signal in cells expressing Mps1^WT^ rescue plasmid, but not in cells expressing Mps1^KD^. Compared with that of cells expressing Mps1^WT^, the pMELT-Knl1 signal is clearly weaker in cells expressing Mps1^ΔCT^ (Figure 4A and B). Similarly, Mps1^WT^-expressing cells display stronger Mad2 kinetochore signal, whereas cells expressing Mps1^ΔCT^ have decreased Mad2 kinetochore signal (Figure 4C and D). A previous publication concluded that Mps1^CT^ is critical for substrate recruitment (Sun et al., 2010). To verify this conclusion, we evaluated the kinase activity of recombinant Mps1^C342^ and Mps1^PK^ through an *in vitro* kinase assay. As determined by anti-pMELT-Knl1 blotting, Mps1^C342^ and Mps1^PK^ have equivalent kinase activity toward GST-Knl1^871–960^ (Supplementary Figure S5A). No signals were detected in the reactions using Mps1^C342-KD^ and Mps1^PK-KD^, proving the specificity of the reaction. Thus, we conclude that Mps1^CT^ is critical for phosphorylating physiological substrates *in vivo*, but not for kinase activity *in vitro*.

We further examined the mitotic progression in the cells expressing Mps1^ΔCT^ by means of live cell imaging. When Mps1 is knocked down, GFP-expressing cells enter anaphase prematurely with the presence of numerous unaligned chromosomes (Supplementary Figure S5B). In cells rescued with Mps1^WT^, the majority of cells finish faithful sister chromosome segregation (Figure 4E; Supplementary Figure S5C). On the contrary, a large proportion of cells expressing Mps1^ΔCT^ suffer erroneous chromosome segregation as indicated by the anaphase lagging chromosomes (Figure 4E; Supplementary Figure S5D). Quantification of the time from nuclear envelope breakdown (NEBD) to anaphase onset indicated that the Mps1^ΔCT^-expressing cells spend shorter time to enter anaphase than Mps1^WT^-expressing cells, even with the unaligned chromosomes (Figure 4F). Taken together, these data suggest that the Mps1 C-tail is critical for the functional integrity of the SAC function and faithful mitotic progression.

## Discussion

As an initiating factor for SAC signaling, the accurate spatiotemporal localization of Mps1 to the kinetochore is critical for SAC function. In light of its leading role in SAC signaling, Mps1 has a unique localization pattern among the several kinases involved in SAC: Mps1 kinase activity substantially alleviates its own kinetochore localization (Pachis and Kops, 2018). Previous publications supported that there are multisite interactions between Mps1 and Ndc80C (Hiruma et al., 2015; Ji et al., 2015). Here, we found that a single Mps1 kinetochore targeting module has a very weak binding affinity with Ndc80C and displays invisible kinetochore localization. With the combination of two modules, the fusion proteins have weak but clear kinetochore localization. Induced dimerization further enhanced Mps1 localization, supporting the fact that dimerization contributes to its targeting to the kinetochore. Ji et al. (2015) showed that the Mps1^NTE^ binds with the Hec1 CH domain and the Mps1 middle region (referred as Mps1^CTE^ in this study) binds with the Nuf2 CH domain. However, they failed to detect the direct interaction between Ndc80C and Mps1^TPR^. In our assays, TPR could easily be pulled down by Ndc80C^Bonsai^. Consistent with the strong Ndc80C and TPR interaction, previous studies demonstrated the importance of TPR in mediating Mps1 kinetochore localization (Thebault et al., 2012; Nijenhuis et al., 2013; Marquardt et al., 2016). Thus, we conclude that the TPR domain is involved in mediating Mps1–Ndc80C interaction directly. Studies from the Kops group concluded that TPR has an inhibitory effect on Mps1 kinetochore localization and NTE–TPR interaction promotes Mps1 release from the kinetochore (Nijenhuis et al., 2013; Pachis et al., 2019). However, in our opinion, the direct biochemical interaction between TPR and Ndc80C supports the fact that the major role of TPR is to mediate Mps1 targeting to the kinetochore, although the NTE does interact with TPR.

Numerous studies support the importance of Aurora B kinase activity in enhancing Mps1 kinetochore recruitment (Vigneron et al., 2004; Santaguida et al., 2010; Dou et al., 2011; Saurin et al., 2011; Nijenhuis et al., 2013; Zhu et al., 2013; Ji et al., 2015). However, due to the presence of complicated kinases signaling wiring such as Cdk1–Tip60–Aurora B axis and Mps1–Mad1–cyclin B1 axis, the underlying mechanism is unclear (Mo et al., 2016; Bao et al., 2018; Alfonso-Perez et al., 2019). We found that Hec1^9D^ has a significantly stronger binding affinity with all three Mps1 kinetochore-binding modules (Figure 1D), supporting Aurora B-elicited phosphorylation toward the Hec1 N-tail relieves the inhibitory effect of Hec1 N-tail toward CH domain of both Hec1 and Nuf2 (Ji et al., 2015; Zhao et al., 2019).

We further found that the Mps1 C-terminal fragment enhances its kinetochore localization through dimerization. Specifically, we identified two short α-helices within the Mps1^CT^ that are essential for promoting Mps1 kinetochore localization. The dimerization of Mps1 has been previously proposed without deep exploration (Kang et al., 2007; Hewitt et al., 2010; Lee et al., 2012; Nijenhuis et al., 2013). Consistent with the observation that Mps1^Δ200^ was readily detectable at kinetochores of cells containing normal levels of endogenous Mps1 (Nijenhuis et al., 2013), we confirmed that Mps1 dimerization is mainly mediated by the Mps1^C342^ fragment. Based on our biochemical analysis, we concluded that Mps1^CT^ can greatly enhance the dimerization of Mps1^C342^ (Supplementary Figure S4E). However, we failed to detect the direct interaction between Mps1^CT^ fragments (Supplementary Figure S4F). We reason that either Mps1^CT^ mediates dimerization together with Mps1^PK-KD^ synergistically or the expression of Mps1^CT^ fails to fold as in its native conformation. Interestingly but not surprisingly, Mps1^C342^-mediated dimerization is negatively regulated by its protein kinase activity. We speculate that the active conformation of protein kinase domain may alleviate the Mps1 dimerization. The other likelihood is that auto-phosphorylation sites within the Mps1^PK^ or Mps1^CT^ preclude the Mps1 dimerization. Consistent with the previous publication (Thebault et al., 2012), our study also supports the idea that Mps1^N300^ has comparable weaker dimerization activity. Taking into account the direct interaction between Mps1^NTE^ and the kinase domain (Combes et al., 2018), we envision that Mps1 protein has complicated intra-molecular N300–N300 and C342–C342 interactions and inter-molecular or intra-molecular N300–C342 interactions.

During mitosis, Mps1 protein was phosphorylated by Cdk1, Plk1, and Mps1 itself. One of the Cdk1 substrate sites is Mps1 S821, which is located within the C-tail. Previous study showed that phosphorylation of *Xenopus* Mps1 S844 (equivalent to S821 of human Mps1) by MAPK is required for Mps1 kinetochore localization (Zhao and Chen, 2006). According to our previous study, Mps1 S821 is most likely a substrate of Cdk1, and S281 is another key substrate of Cdk1 (Dou et al., 2011). The current understanding of S281 phosphorylation remains controversial: whether S281 phosphorylation is critical for Mps1 kinetochore localization is still on debate (Morin et al., 2012; Hayward et al., 2019). The roles of S281 and S821 phosphorylation need to be dissected carefully in the future. Very recently, a study found that Mps1 autophosphorylation is sufficient to release itself from the kinetochore in yeast, supporting our previous finding that human Mps1 autophosphorylations promote its release from the kinetochore (Wang et al., 2014; Koch et al., 2019). It is necessary to test the roles of specific autophosphorylation sites and other posttranslational modifications such as SUMOylation in future studies (Restuccia et al., 2016). Although the role of human Mps1 in centrosome duplication is controversial (Stucke et al., 2002; Fisk et al., 2003; Kwiatkowski et al., 2010), given the fact that centrosome and spindle pole are key mitotic apparatus, it is worth to evaluate the potential involvement of centrosome and spindle pole-associated Mps1 in the checkpoint activation/inactivation and mitotic progression.

**Figure 5:**
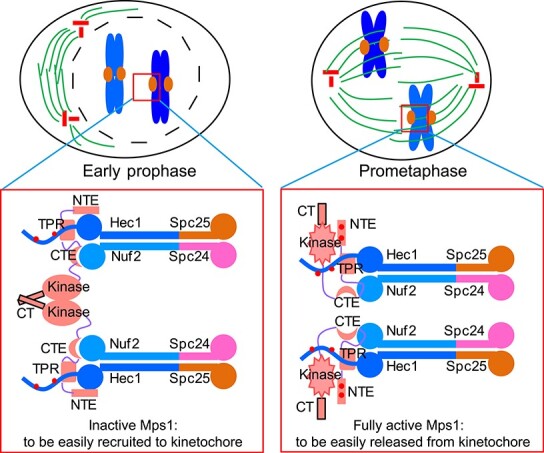
The model of dynamic Mps1 kinetochore localization. During prophase, the majority of Mps1 molecules are in an inactive form. Once the outer kinetochore Knl1–Mis12–Ndc80 network assembles, inactive Mps1 molecules are recruited to the kinetochore through the multisite interactions with Ndc80C. Mps1 dimerization further enhances its binding affinity with Ndc80C. The high affinity of kinetochore recruitment of Mps1 enables its fast activation via autophosphorylation *in trans*. During prometaphase, active Mps1 molecules bind to the kinetochore with a lower affinity due to weakened dimerization and autophosphorylation. The low affinity of kinetochore targeting of active Mps1 allows the establishment of microtubule attachment and efficient SAC signaling silence. Spc24, spindle pole body component 24; Spc25, spindle pole body component 25.

Consistent with a previous report (Sun et al., 2010), our functional studies demonstrated that deleting Mps1^CT^ compromises SAC signaling and causes an elevated level of chromosomal segregation defects. We envision that Mps1^CT^ contributes to the quick kinetochore recruitment of Mps1 before its full activation and therefore the timely establishment of SAC signaling once cells entered prometaphase. Our work favors a model that multisite interactions, dimerization, and autophosphorylation work together to contribute to the spatiotemporal dynamics of Mps1 kinetochore localization (Figure 5). Before full activation (early prophase), Mps1^N300^-mediated multisite interactions with Ndc80C and Mps1^C342^-mediated dimerization permit high-affinity localization of Mps1 to the kinetochore. Once activated, Mps1 autophosphorylation reduces the binding affinity between Mps1 and Ndc80C (Wang et al., 2014; Koch et al., 2019). Therefore, active Mps1 localizes at unattached or improperly attached kinetochores with a high turnover. This high turnover localization allows microtubules to compete with Mps1 to bind to Ndc80C (Hiruma et al., 2015; Ji et al., 2015). Consequently, Mps1 is released from the kinetochore, and SAC signaling is satisfied. Artificially tethering Mps1 at the kinetochore through Mis12–Mps1 fusion protein causes mitotic arrest, arguing that the release of Mps1 from the kinetochore is a key precondition for responsive SAC signaling silencing (Jelluma et al., 2010). Previously, our group also demonstrated that the dynamic localization of Mps1 to the kinetochore is essential for accurate spindle microtubule attachment (Dou et al., 2015). It would be of great interest, down the road, to model Mps1 function using recently established 3D organoids model combined with chemical biological tools (Drost and Clevers, 2018; Liu et al., 2019; Yao and Smolka, 2019), which will unravel the context-dependent function of Mps1 such as pathogenesis of solid tumors.

In summary, the multisite biochemical binding of Mps1 to Ndc80C and Mps1 dimerization and autophosphorylation endow Mps1 to be easily recruited to the kinetochore before activation and to be easily released from the kinetochore after activation. This dynamic kinetochore recruitment is critical for the functional integrity of SAC signaling. This study advances our understanding of the dynamic kinetochore recruitment of Mps1 by Ndc80C and provides new insights on responsive SAC signaling.

## Materials and methods

### Cell culture and drug concentration

HeLa cells were routinely maintained in DMEM (Invitrogen) supplemented with 10% fetal bovine serum (FBS) and penicillin-streptomycin (100 IU/ml and 100 mg/ml, respectively; Gibco). U2OS-LacO cells were cultured in DMEM (Invitrogen) supplemented with 10% FBS and penicillin-streptomycin plus hygromycin (100 μg/ml; Sigma). BAC TransgeneOmics LAP–Mps1 stable HeLa cells were kindly provided by A. Hyman (Max Planck Institute, Dresden, Germany) and were maintained in DMEM containing G418 (0.5 μg/μl).

Thymidine was used at 2 mM, nocodazole at 100 ng/ml, Mps1 inhibitor reversine at 0.5 μM, and MG132 at 20 μM. For chemical-induced dimerization, cells were treated with AP20187 (B/B Homodimerizer) at 10 nM for 30 min.

### RNA interference and transfection

All the expression plasmids and shRNA plasmids were transfected into cells using Lipofectamine 2000 (Invitrogen) according to user’s manual. To enrich mitotic cells, cells were treated at 10 h after transfection with thymidine for 14–16 h. Then cells were released into normal DMEM medium. After 8 h, cells were treated with nocodazole for 2 h and then fixed for immunofluorescence staining. Mps1 shRNA plasmid pSuper-Mps1 (shMps1) was described previously (Jelluma et al., 2008). For rescue experiments, Mps1 shRNA was co-transfected with different rescue plasmids (or empty vector) at a ratio of 3:1.

### Antibodies

Mouse anti-hMps1-N1 (Abcam, Ab11108, 1:500), mouse anti-Mad2 (CM2^276^, Santa Cruz, Sc-65492, 1:200), mouse anti-α-tubulin (Cell Signaling Technology, DM1A, 3873, 1:5000), mouse anti-MBP (Cell Signaling Technology, 8G1, 2396, 1:2000), rabbit anti-GFP (Proteintech, 50430-2-AP, 1:1000), mouse anti-His-tag (Cell Signaling Technology, 27E8, 2366, 1:2000), mouse anti-Flag (Sigma, F3165, 1:2000), and human anti-centromere auto-antibody (ACA, Immunovision, HCT-0100, 1:5000) were obtained commercially. Anti-pMELT-Knl1 antibody was kindly gifted by Dr Geert Kops (Hubrecht Institute, the Netherlands) (Nijenhuis et al., 2014).

### Live cell imaging

HeLa cells were cultured in glass-bottomed culture dishes (MatTek). Cells were co-transfected with shMps1, different shMps1-resistent GFP-Mps1 rescue plasmids, and mCherry-H2B at a ratio of 6:2:1. After 36 h, cells were cultured at 37°C in CO_2_-independent medium (Invitrogen) containing 10% FBS and 2 mM glutamine and observed with the DeltaVision RT system (Applied Precision) as previously described (Akram et al., 2018). Images were prepared for publication using Adobe Photoshop software.

## Supplementary Material

JMCB-2019-0409_R2_Supplementary_Material_mjaa006

## References

[ref1] Akram, S., Yang, F., Li, J., et al. (2018). LRIF1 interacts with HP1α to coordinate accurate chromosome segregation during mitosis. J. Mol. Cell Biol. 10, 527–538. 30016453 10.1093/jmcb/mjy040 PMC6304163

[ref2] Alfonso-Perez, T., Hayward, D., Holder, J., et al. (2019). MAD1-dependent recruitment of CDK1–CCNB1 to kinetochores promotes spindle checkpoint signaling. J. Cell Biol. 218, 1108–1117. 30674583 10.1083/jcb.201808015 PMC6446853

[ref3] Bao, X., Liu, H., Liu, X., et al. (2018). Mitosis-specific acetylation tunes Ran effector binding for chromosome segregation. J. Mol. Cell Biol. 10, 18–32. 29040603 10.1093/jmcb/mjx045 PMC6041754

[ref4] Cheeseman, I.M., Chappie, J.S., Wilson-Kubalek, E.M., et al. (2006). The conserved KMN network constitutes the core microtubule-binding site of the kinetochore. Cell 127, 983–997. 17129783 10.1016/j.cell.2006.09.039

[ref5] Ciferri, C., Pasqualato, S., Screpanti, E., et al. (2008). Implications for kinetochore–microtubule attachment from the structure of an engineered Ndc80 complex. Cell 133, 427–439. 18455984 10.1016/j.cell.2008.03.020 PMC4754795

[ref6] Combes, G., Barysz, H., Garand, C., et al. (2018). Mps1 phosphorylates its N-terminal extension to relieve autoinhibition and activate the spindle assembly checkpoint. Curr. Biol. 28, 872–883.e5. 29502948 10.1016/j.cub.2018.02.002 PMC5863767

[ref7] DeLuca, J.G., Gall, W.E., Ciferri, C., et al. (2006). Kinetochore microtubule dynamics and attachment stability are regulated by Hec1. Cell 127, 969–982. 17129782 10.1016/j.cell.2006.09.047

[ref8] Dou, Z., Liu, X., Wang, W., et al. (2015). Dynamic localization of Mps1 kinase to kinetochores is essential for accurate spindle microtubule attachment. Proc. Natl Acad. Sci. USA 112, E4546–E4555. 26240331 10.1073/pnas.1508791112 PMC4547264

[ref9] Dou, Z., Prifti, D.K., Gui, P., et al. (2019). Recent progress on the localization of the spindle assembly checkpoint machinery to kinetochores. Cells 8, 278. 10.3390/cells8030278 PMC6468716 30909555

[ref10] Dou, Z., von Schubert, C., Korner, R., et al. (2011). Quantitative mass spectrometry analysis reveals similar substrate consensus motif for human Mps1 kinase and Plk1. PLoS One 6, e18793. 21533207 10.1371/journal.pone.0018793 PMC3076450

[ref11] Drost, J., and Clevers, H. (2018). Organoids in cancer research. Nat. Rev. Cancer 18, 407–418. 29692415 10.1038/s41568-018-0007-6

[ref12] Fisk, H.A., Mattison, C.P., and Winey, M. (2003). Human Mps1 protein kinase is required for centrosome duplication and normal mitotic progression. Proc. Natl Acad. Sci. USA 100, 14875–14880. 14657364 10.1073/pnas.2434156100 PMC299837

[ref13] Hayward, D., Alfonso-Perez, T., and Gruneberg, U. (2019). Orchestration of the spindle assembly checkpoint by CDK1–cyclin B1. FEBS Lett. 593, 2889–2907. 31469407 10.1002/1873-3468.13591

[ref14] Heinrich, S., Windecker, H., Hustedt, N., et al. (2012). Mph1 kinetochore localization is crucial and upstream in the hierarchy of spindle assembly checkpoint protein recruitment to kinetochores. J. Cell Sci. 125, 4720–4727. 22825872 10.1242/jcs.110387

[ref15] Hewitt, L., Tighe, A., Santaguida, S., et al. (2010). Sustained Mps1 activity is required in mitosis to recruit O-Mad2 to the Mad1–C-Mad2 core complex. J. Cell Biol. 190, 25–34. 20624899 10.1083/jcb.201002133 PMC2911659

[ref16] Hiruma, Y., Sacristan, C., Pachis, S.T., et al. (2015). Competition between MPS1 and microtubules at kinetochores regulates spindle checkpoint signaling. Science 348, 1264–1267. 26068855 10.1126/science.aaa4055

[ref17] Huang, Y., Lin, L., Liu, X., et al. (2019). BubR1 phosphorylates CENP-E as a switch enabling the transition from lateral association to end-on capture of spindle microtubules. Cell Res. 29, 562–578. 31201382 10.1038/s41422-019-0178-z PMC6796941

[ref18] Janicki, S.M., Tsukamoto, T., Salghetti, S.E., et al. (2004). From silencing to gene expression: real-time analysis in single cells. Cell 116, 683–698. 15006351 10.1016/s0092-8674(04)00171-0 PMC4942132

[ref19] Jelluma, N., Brenkman, A.B., van den Broek, N.J., et al. (2008). Mps1 phosphorylates Borealin to control Aurora B activity and chromosome alignment. Cell 132, 233–246. 18243099 10.1016/j.cell.2007.11.046

[ref20] Jelluma, N., Dansen, T.B., Sliedrecht, T., et al. (2010). Release of Mps1 from kinetochores is crucial for timely anaphase onset. J. Cell Biol. 191, 281–290. 20937696 10.1083/jcb.201003038 PMC2958484

[ref21] Ji, Z., Gao, H., and Yu, H. (2015). Kinetochore attachment sensed by competitive Mps1 and microtubule binding to Ndc80C. Science 348, 1260–1264. 26068854 10.1126/science.aaa4029

[ref22] Kang, J., Chen, Y., Zhao, Y., et al. (2007). Autophosphorylation-dependent activation of human Mps1 is required for the spindle checkpoint. Proc. Natl Acad. Sci. USA 104, 20232–20237. 18083840 10.1073/pnas.0710519105 PMC2154414

[ref23] Koch, L.B., Opoku, K.N., Deng, Y., et al. (2019). Autophosphorylation is sufficient to release Mps1 kinase from native kinetochores. Proc. Natl Acad. Sci. USA 116, 17355–17360. 31405987 10.1073/pnas.1901653116 PMC6717314

[ref24] Kwiatkowski, N., Jelluma, N., Filippakopoulos, P., et al. (2010). Small-molecule kinase inhibitors provide insight into Mps1 cell cycle function. Nat. Chem. Biol. 6, 359–368. 20383151 10.1038/nchembio.345 PMC2857554

[ref25] Lavoie, H., Li, J.J., Thevakumaran, N., et al. (2014). Dimerization-induced allostery in protein kinase regulation. Trends Biochem. Sci. 39, 475–486. 25220378 10.1016/j.tibs.2014.08.004

[ref26] Lee, S., Thebault, P., Freschi, L., et al. (2012). Characterization of spindle checkpoint kinase Mps1 reveals domain with functional and structural similarities to tetratricopeptide repeat motifs of Bub1 and BubR1 checkpoint kinases. J. Biol. Chem. 287, 5988–6001. 22187426 10.1074/jbc.M111.307355 PMC3285366

[ref27] Liu, S.T., Chan, G.K., Hittle, J.C., et al. (2003). Human MPS1 kinase is required for mitotic arrest induced by the loss of CENP-E from kinetochores. Mol. Biol. Cell 14, 1638–1651. 12686615 10.1091/mbc.02-05-0074 PMC153128

[ref28] Liu, X., Xu, L., Li, J., et al. (2019). Mitotic motor CENP-E cooperates with PRC1 in temporal control of central spindle assembly. J. Mol. Cell Biol. doi: 10.1093/jmcb/mjz051. PMC7683015 31174204

[ref29] London, N., and Biggins, S. (2014). Signalling dynamics in the spindle checkpoint response. Nat. Rev. Mol. Cell Biol. 15, 736–747. 25303117 10.1038/nrm3888 PMC4283840

[ref30] Luo, Y., Ahmad, E., and Liu, S.T. (2018). MAD1: kinetochore receptors and catalytic mechanisms. Front. Cell Dev. Biol. 6, 51. 29868582 10.3389/fcell.2018.00051 PMC5949338

[ref31] Maciejowski, J., Drechsler, H., Grundner-Culemann, K., et al. (2017). Mps1 regulates kinetochore–microtubule attachment stability via the Ska complex to ensure error-free chromosome segregation. Dev. Cell 41, 143–156.e6. 28441529 10.1016/j.devcel.2017.03.025 PMC5477644

[ref32] Marquardt, J.R., Perkins, J.L., Beuoy, K.J., et al. (2016). Modular elements of the TPR domain in the Mps1 N terminus differentially target Mps1 to the centrosome and kinetochore. Proc. Natl Acad. Sci. USA 113, 7828–7833. 27339139 10.1073/pnas.1607421113 PMC4948323

[ref33] Martin-Lluesma, S., Stucke, V.M., and Nigg, E.A. (2002). Role of Hec1 in spindle checkpoint signaling and kinetochore recruitment of Mad1/Mad2. Science 297, 2267–2270. 12351790 10.1126/science.1075596

[ref34] Maure, J.F., Kitamura, E., and Tanaka, T.U. (2007). Mps1 kinase promotes sister-kinetochore bi-orientation by a tension-dependent mechanism. Curr. Biol. 17, 2175–2182. 18060784 10.1016/j.cub.2007.11.032 PMC2515371

[ref35] Mo, F., Zhuang, X., Liu, X., et al. (2016). Acetylation of Aurora B by TIP60 ensures accurate chromosomal segregation. Nat. Chem. Biol. 12, 226–232. 26829474 10.1038/nchembio.2017 PMC4798883

[ref36] Moore, A., and Wordeman, L. (2004). The mechanism, function and regulation of depolymerizing kinesins during mitosis. Trends Cell Biol. 14, 537–546. 15450976 10.1016/j.tcb.2004.09.001

[ref37] Morin, V., Prieto, S., Melines, S., et al. (2012). CDK-dependent potentiation of MPS1 kinase activity is essential to the mitotic checkpoint. Curr. Biol. 22, 289–295. 22245000 10.1016/j.cub.2011.12.048

[ref38] Musacchio, A. (2015). The molecular biology of spindle assembly checkpoint signaling dynamics. Curr. Biol. 25, R1002–R1018. 26485365 10.1016/j.cub.2015.08.051

[ref39] Nijenhuis, W., Vallardi, G., Teixeira, A., et al. (2014). Negative feedback at kinetochores underlies a responsive spindle checkpoint signal. Nat. Cell Biol. 16, 1257–1264. 25402682 10.1038/ncb3065 PMC6485516

[ref40] Nijenhuis, W., von Castelmur, E., Littler, D., et al. (2013). A TPR domain-containing N-terminal module of MPS1 is required for its kinetochore localization by Aurora B. J. Cell Biol. 201, 217–231. 23569217 10.1083/jcb.201210033 PMC3628519

[ref41] Overlack, K., Primorac, I., Vleugel, M., et al. (2015). A molecular basis for the differential roles of Bub1 and BubR1 in the spindle assembly checkpoint. eLife 4, e05269. 25611342 10.7554/eLife.05269 PMC4337726

[ref42] Pachis, S.T., Hiruma, Y., Tromer, E.C., et al. (2019). Interactions between N-terminal modules in MPS1 enable spindle checkpoint silencing. Cell Rep. 26, e2106. 10.1016/j.celrep.2019.01.017 30784592

[ref43] Pachis, S.T., and Kops, G. (2018). Leader of the SAC: molecular mechanisms of Mps1/TTK regulation in mitosis. Open Biol. 8, 180109. 30111590 10.1098/rsob.180109 PMC6119859

[ref44] Restuccia, A., Yang, F., Chen, C., et al. (2016). Mps1 is SUMO-modified during the cell cycle. Oncotarget 7, 3158–3170. 26675261 10.18632/oncotarget.6552 PMC4823097

[ref45] Santaguida, S., and Amon, A. (2015). Short- and long-term effects of chromosome mis-segregation and aneuploidy. Nat. Rev. Mol. Cell Biol. 16, 473–485. 26204159 10.1038/nrm4025

[ref46] Santaguida, S., Tighe, A., D'Alise, A.M., et al. (2010). Dissecting the role of MPS1 in chromosome biorientation and the spindle checkpoint through the small molecule inhibitor reversine. J. Cell Biol. 190, 73–87. 20624901 10.1083/jcb.201001036 PMC2911657

[ref47] Santaguida, S., Vernieri, C., Villa, F., et al. (2011). Evidence that Aurora B is implicated in spindle checkpoint signalling independently of error correction. EMBO J. 30, 1508–1519. 21407176 10.1038/emboj.2011.70 PMC3102279

[ref48] Saurin, A.T., van der Waal, M.S., Medema, R.H., et al. (2011). Aurora B potentiates Mps1 activation to ensure rapid checkpoint establishment at the onset of mitosis. Nat. Commun. 2, 316. 21587233 10.1038/ncomms1319 PMC3113227

[ref49] Stucke, V.M., Sillje, H.H., Arnaud, L., et al. (2002). Human Mps1 kinase is required for the spindle assembly checkpoint but not for centrosome duplication. EMBO J. 21, 1723–1732. 11927556 10.1093/emboj/21.7.1723 PMC125937

[ref50] Sun, T., Yang, X., Wang, W., et al. (2010). Cellular abundance of Mps1 and the role of its carboxyl terminal tail in substrate recruitment. J. Biol. Chem. 285, 38730–38739. 20884615 10.1074/jbc.M110.177642 PMC2992306

[ref51] Suzuki, A., and Varma, D. (2018). Cell division: the unattached kinetochore wears an expansive RZZ coat. Curr. Biol. 28, R1250–R1252. 30399347 10.1016/j.cub.2018.10.001

[ref52] Thebault, P., Chirgadze, D.Y., Dou, Z., et al. (2012). Structural and functional insights into the role of the N-terminal Mps1 TPR domain in the SAC (spindle assembly checkpoint). Biochem. J. 448, 321–328. 23067341 10.1042/BJ20121448

[ref53] Ubersax, J.A., and Ferrell, J.E., Jr. (2007). Mechanisms of specificity in protein phosphorylation. Nat. Rev. Mol. Cell Biol. 8, 530–541. 17585314 10.1038/nrm2203

[ref54] Vigneron, S., Prieto, S., Bernis, C., et al. (2004). Kinetochore localization of spindle checkpoint proteins: who controls whom? Mol. Biol. Cell 15, 4584–4596. 15269280 10.1091/mbc.E04-01-0051 PMC519151

[ref55] Wang, X., Yu, H., Xu, L., et al. (2014). Dynamic autophosphorylation of mps1 kinase is required for faithful mitotic progression. PLoS One 9, e104723. 25265012 10.1371/journal.pone.0104723 PMC4179234

[ref56] Watson, E.R., Brown, N.G., Peters, J.M., et al. (2019). Posing the APC/C E3 ubiquitin ligase to orchestrate cell division. Trends Cell Biol. 29, 117–134. 30482618 10.1016/j.tcb.2018.09.007 PMC6340778

[ref57] Xia, P., Liu, X., Wu, B., et al. (2014). Superresolution imaging reveals structural features of EB1 in microtubule plus-end tracking. Mol. Biol. Cell 25, 4166–4173. 25355949 10.1091/mbc.E14-06-1133 PMC4263457

[ref58] Yao, X., and Smolka, A.J. (2019). Gastric parietal cell physiology and helicobacter pylori-induced disease. Gastroenterology 156, 2158–2173. 30831083 10.1053/j.gastro.2019.02.036 PMC6715393

[ref59] Zhang, G., Lischetti, T., Hayward, D.G., et al. (2015). Distinct domains in Bub1 localize RZZ and BubR1 to kinetochores to regulate the checkpoint. Nat. Commun. 6, 7162. 26031201 10.1038/ncomms8162 PMC4458899

[ref60] Zhao, G., Cheng, Y., Gui, P., et al. (2019). Dynamic acetylation of the kinetochore-associated protein HEC1 ensures accurate microtubule–kinetochore attachment. J. Biol. Chem. 294, 576–592. 30409912 10.1074/jbc.RA118.003844 PMC6333894

[ref61] Zhao, Y., and Chen, R.H. (2006). Mps1 phosphorylation by MAP kinase is required for kinetochore localization of spindle-checkpoint proteins. Curr. Biol. 16, 1764–1769. 16950116 10.1016/j.cub.2006.07.058

[ref62] Zhu, T., Dou, Z., Qin, B., et al. (2013). Phosphorylation of microtubule-binding protein Hec1 by mitotic kinase Aurora B specifies spindle checkpoint kinase Mps1 signaling at the kinetochore. J. Biol. Chem. 288, 36149–36159. 24187132 10.1074/jbc.M113.507970 PMC3861662

