# Supplementary material for: Mps1 dimerization and multisite interactions with Ndc80 complex enable responsive spindle assembly checkpoint signaling
Source: J Mol Cell Biol. 2020 Mar 26;12(7):486–98. doi: 10.1093/jmcb/mjaa006 (PMC7493027; doi:10.1093/jmcb/mjaa006)
Supplement: JMCB-2019-0409_R2_Supplementary_Material_mjaa006 [file jmcb-2019-0409_r2_supplementary_material_mjaa006.pdf]

Supplementary Materials and Methods

Plasmids and Mutagenesis

PrimeSTAR DNA polymerase (Takara Biomedical Technology) was used to amplify different cDNA fragments. GFP-tagged full-length Mps1 and different Mps1 truncations were generated by inserting the corresponding PCR-amplified fragments into pEGFP-C1 vector via BglII and SalI sites. GFP-tagged Mps1 truncations construct $Mps1^{NTE\text{-}CTE}$ was generated by inserting $Mps1^{192\text{-}300}$ PCR-amplified fragments into GFP-$Mps1^{1\text{-}61}$ plasmid via SalI and BamHI sites. GFP-$Mps1^{N300\text{-}FKBP}$ plasmid was generated by inserting FKBP12 PCR-amplified fragment into GFP-$Mps1^{N300}$ via BamHI site. For different constructs expressing Maltose binding protein (MBP) tagged Mps1 truncation proteins with a C-terminal 6×His-tag, different PCR-amplified Mps1 fragments were inserted into the pET30a-MBP expression vector (a gift from Prof. Tengchuan Jin, USTC, China) by NotI and XhoI. LAP-Mps1 plasmid is a gift from Prof. Geert J.P.L. Kops. (Hubrecht institute, the Netherlands). GFP-ZW10 plasmid was described previously (Wang et al., 2004).

For LacO-LacI ectopic recruitment experiments, different pmCherry-LacI-Mps1 plasmids were generated by sequentially inserting LacI cDNA into pmCherry-C2 vector using BglII and SalI sites and insert different Mps1 cDNA using SalI and BamHI sites. For bimolecular fluorescence complementation experiments, different Mps1 fragments were inserted into a vector expressing N-terminal $EGFP^{1\text{-}155}$ fragment and another vector expressing N-terminal $EGFP^{156\text{-}238}$ fragment.

Mutagenesis was performed using Mut Express II fast mutagenesis kit (Vazyme Biotech Co. Ltd) according to the manufacturer's instructions. All constructs were verified by sequencing.

Immunofluorescence microscopy, image processing and quantification

Unless indicated specifically, HeLa cells grown on coverslips were fixed and permeabilized simultaneously with PTEMF buffer (50 mM PIPES, pH6.8, 0.2% Triton X-100, 10 mM EGTA, 1 mM $MgCl_2$, 3.7% Formaldehyde) at room temperature and processed for indirect immunofluorescence microscopy. Samples were examined on a DeltaVision microscope (GE Life Sciences), with optical sections acquired 0.25 μm apart in the Z-axis. Deconvolved images from each focal plane were projected into a single picture using Softworx (GE Life Sciences). Images were taken at identical exposure times within each experiment, acquired as 8-bit gray scale images. After deconvolution, the images were exported as 24-bit RGB images and processed in Adobe Photoshop. Images shown in the same panel have been identically scaled. Measurement of kinetochore intensities was performed in ImageJ on non-deconvolved images. Quantification of the level of kinetochore-associated protein was conducted as described previously (Dou et al., 2015). In brief, the average pixel intensities from at least 20 kinetochore pairs from each cell were measured, and background pixel intensities were subtracted. The pixel intensities at each kinetochore pair were then normalized against ACA pixel values to account for any variations in staining or image acquisition. In all plots, each dot represents one cell. All *p*-values were calculated using the Student's *t*-test with the Prism software (GraphPad).

Western Blot

Samples were separated by standard SDS-PAGE electrophoresis, then transferred to nitrocellulose membranes. Western blotting was performed using standard protocols. For all Western blotting, signals were detected using HRP-conjugated anti-mouse or anti-rabbit antibodies (Pierce) through an Amersham Imager 600 machine.

Recombinant protein expression and GST pull-down assay

Different glutathione S-transferase (GST) fusion proteins were expressed in bacteria BL21-Gold(DE3) strain and purified by using glutathione agaroseas reported previously (Zhao et al., 2019). GST-Ndc80^{Bonsai}, and GST-Ndc80$^{Bonsai\Delta N}$ fusion proteins were purified as previously described (Ciferri et al., 2008). Different MBP-Mps1-His_6 fusion proteins were purified using Ni-NTA beads (Qiagen) according to manufacturer's protocol. GST-tagged Ndc80^{Bonsai} or Ndc80$^{Bonsai\Delta N}$ fusion protein-bound glutathione beads were incubated with purified and eluted MBP-Mps1-His_6 fusion proteins in PBS buffer containing 0.1% Triton X-100 plus 1 mM PMSF and 1 mM DTT for 1 hour at 4°C. After the incubation, the beads were washed 3 times with PBS containing 0.1% Triton X-100 and once with PBS and boiled in SDS-PAGE sample buffer. The bound proteins were then separated on 10% SDS-PAGE. In parallel, the samples were analyzed by anti-MBP antibody using Western blot.

Supplementary reference

Ciferri, C., Pasqualato, S., Screpanti, E., et al. (2008). Implications for kinetochore-microtubule attachment from the structure of an engineered Ndc80 complex. Cell *133*, 427-439.

Dou, Z., Liu, X., Wang, W., et al. (2015). Dynamic localization of Mps1 kinase to kinetochores is essential for accurate spindle microtubule attachment. Proc Natl Acad Sci U S A *112*, E4546-4555.

Wang, H., Hu, X., Ding, X., et al. (2004). Human Zwint-1 specifies localization of Zeste White 10 to kinetochores and is essential for mitotic checkpoint signaling. J Biol Chem *279*, 54590-54598.

Zhao, G., Cheng, Y., Gui, P., et al. (2019). Dynamic acetylation of the kinetochore-associated protein HEC1 ensures accurate microtubule-kinetochore attachment. J Biol Chem *294*, 576-592.

Figure S1

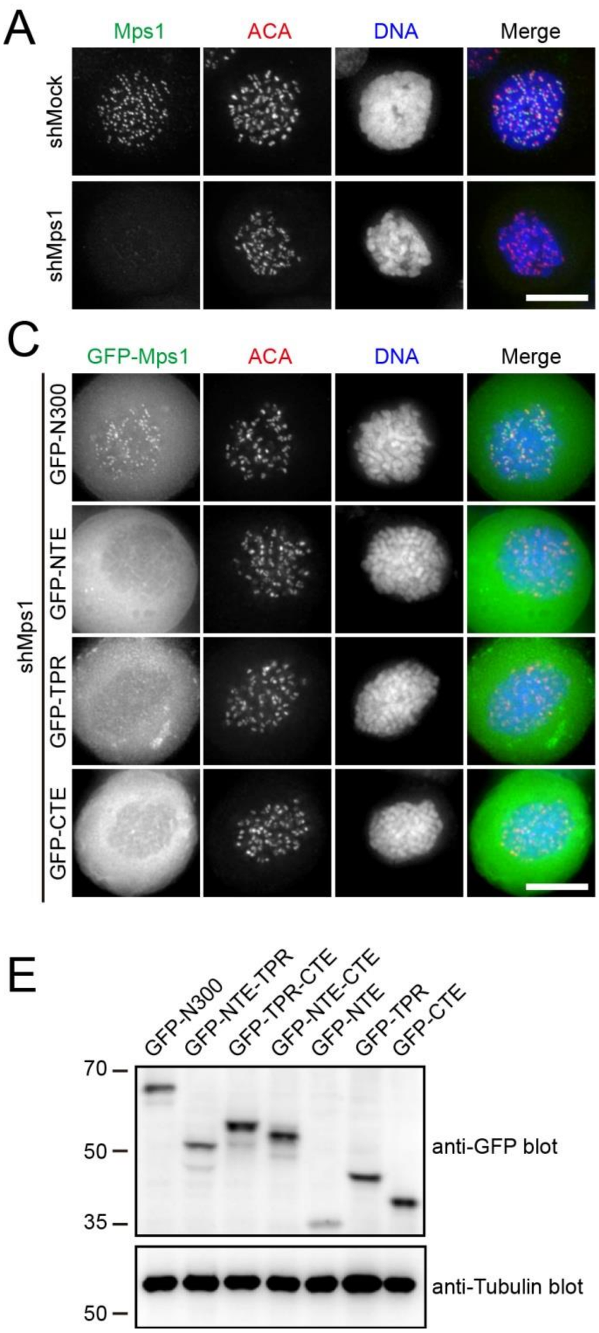

A
Mps1
ACA
DNA
Merge
shMock
shMps1
B
p<0.0001
Mps1 KT signal intensity
Relative intensity
1.5
1.0
0.5
shMock
shMps1
C
GFP-Mps1
ACA
DNA
Merge
shMps1
GFP-N300
GFP-NTE
GFP-TPR
GFP-CTE
D
p<0.0001
Mps1 KT signal intensity
Relative intensity
2.0
1.0
N300
NTE
TPR
CTE
E
GFP-N300
GFP-NTE-TPR
GFP-TPR-CTE
GFP-NTE-CTE
GFP-NTE
GFP-TPR
GFP-CTE
70
50
35
anti-GFP blot
anti-Tubulin blot
50

Figure S1. Individual Mps1 kinetochore localization module fails to localize to kinetochore.

(A) Representative immunofluorescence images of HeLa cells transfected with Mps1 shRNA or Mock shRNA. 36 hours post transfection, cells were treated with Nocodazole plus MG132 for 2 hours to enrich mitotic cells. Then cells were rinsed for 1 min with PHEM buffer (100 mM PIPES, 20 mM HEPES, pH 6.9, 5 mM EGTA, 2 mM $MgCl_2$, and 4 M glycerol) and permeabilized for 1 min with PHEM buffer plus 0.1% Triton X-100. Extracted cells were then fixed in 3.7% formaldehyde in PHEM buffer and rinsed three times in PBS. Afterwards, cells were co-stained with ACA (red), Mps1 (green) and DNA (blue). Scale bar represents 10 μm.

(B) Bar graph illustrating kinetochore intensity of Mps1 protein in cells treated as in A. Bars represent the mean kinetochore intensity (±SD) normalized to the values of shMock group (>30 cells from 3 independent experiments). Student's *t* test was used to calculate *p*-values.

(C, D) Representative images (C) and quantification (D) of HeLa cells expressing indicated GFP-Mps1 truncations. Before fixation, cells were treated with Nocodazole plus MG132 for 2 hours. Then cells were co-stained for ACA (red) and DNA (blue). Scale bar represents 10 μm. Bars represent the mean kinetochore intensity (±SD) normalized to the values of GFP-Mps1-N300 group (>30 cells from 3 independent experiments). Student's *t* test was used to calculate *p*-values.

(E) Western blot showing the comparable expression of different Mps1 constructs as indicated. 24 hours after transfection into HeLa cells, cell lysates were prepared. After separation by SDS-PAGE, samples were probed with the indicated antibodies.

Figure S2

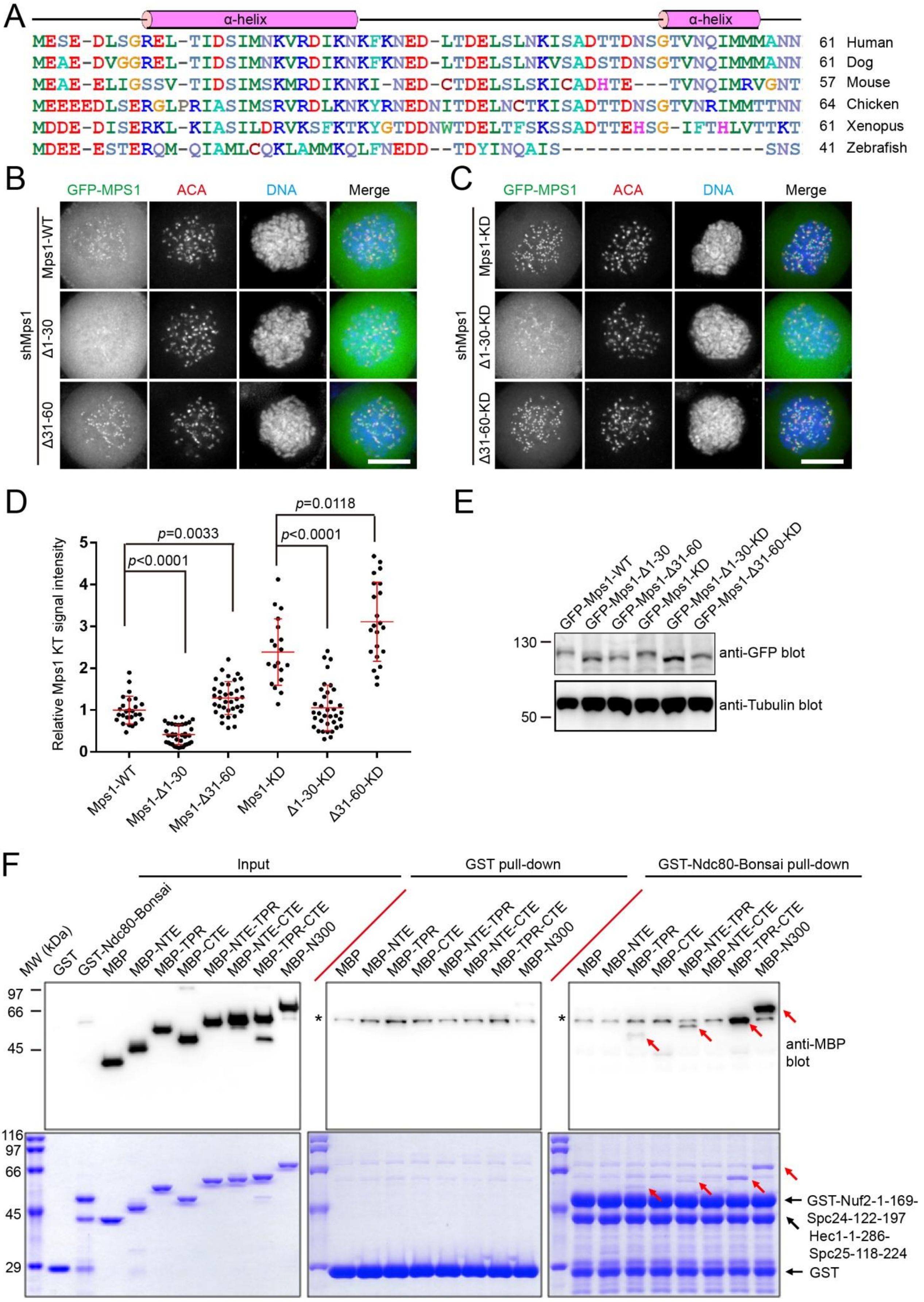

A
α-helix
α-helix
MESE-DLSGREL-TIDSIMNKVRDIKNKFKNED-LTDELSLNKISADTTDNSGTVNQIMMMANN 61 Human
MEAE-DVGGREL-TIDSIMNKVRDIKNKFKNED-LTDELSLNKVSADSTDNSGTVNQIMMMANN 61 Dog
MEAE-ELIGSSV-TIDSIMSKMRDIKNKI-NED-CTDELSLSKICADHTE---TVNQIMRVGNT 57 Mouse
MEEEEDLSERGLPRIASIMSRVRDLKNKYRNEDNITDELNCTKISADTTDNSGTVNRIMMTTNN 64 Chicken
MDDE-DISERKL-KIASILDRVKSFKTKYGTDDNWTDELTFSKSSADTTEHSG-IFTHLVTTKT 61 Xenopus
MDEE-ESTERQM-QIAMLCQKLAMMKQLFNEDD--TDYINQAIS-----------------SNS 41 Zebrafish
B
GFP-MPS1
ACA
DNA
Merge
shMps1
Mps1-WT
Δ1-30
Δ31-60
C
GFP-MPS1
ACA
DNA
Merge
shMps1
Mps1-KD
Δ1-30-KD
Δ31-60-KD
D
Relative Mps1 KT signal intensity
p<0.0001
p=0.0033
p<0.0001
p=0.0118
Mps1-WT
Mps1-Δ1-30
Mps1-Δ31-60
Mps1-KD
Δ1-30-KD
Δ31-60-KD
E
GFP-Mps1-WT
GFP-Mps1-Δ1-30
GFP-Mps1-Δ31-60
GFP-Mps1-KD
GFP-Mps1-Δ1-30-KD
GFP-Mps1-Δ31-60-KD
130
50
anti-GFP blot
anti-Tubulin blot
F
Input
GST pull-down
GST-Ndc80-Bonsai pull-down
MW (kDa)
GST
GST-Ndc80-Bonsai
MBP
MBP-NTE
MBP-TPR
MBP-CTE
MBP-NTE-TPR
MBP-NTE-CTE
MBP-TPR-CTE
MBP-N300
97
66
45
116
29
anti-MBP blot
GST-Nuf2-1-169-Spc24-122-197
Hec1-1-286-Spc25-118-224
GST

Figure S2. The Multisite interactions between Mps1 and Ndc80C.

(A) Multiple sequence alignment of the NTE region of Mps1 proteins from different species as indicated. The sequence alignment was done by ClustalW2 software. The secondary structure was predicted using PSIPRED online tool (http://bioinf.cs.ucl.ac.uk/psipred/).

(B, C) Representative immunofluorescence images of HeLa cells transfected with shMps1 and different shMps1 resistant GFP-Mps1 constructs as indicated. 36 hours post transfection, cells were treated with Nocodazole plus MG132 for 1 hour. Cells were then fixed and co-stained for ACA (red) and DNA (blue). Scale bar represents 10 μm.

(D) Bar graph illustrating kinetochore intensity of different GFP-Mps1 proteins treated as in B and C. Bars represent the mean kinetochore intensity (±SD) normalized to the values of GFP-Mps1-WT group (>30 cells from 3 independent experiments). Student's *t* test was used to calculate *p*-values.

(E) Western blot showing the comparable expression of different Mps1 constructs as indicated. 24 hours after transfection into HeLa cells, cell lysates were prepared. After separation by SDS-PAGE, samples were probed with the indicated antibodies.

(F) GST- or GST-Ndc80^{Bonsai}-bound agarose beads were used as affinity matrices to absorb different MBP-tagged Mps1 truncation proteins purified from E. coli. Pull-downs were analyzed by SDS-PAGE and probed by anti-MBP blotting.

Figure S3

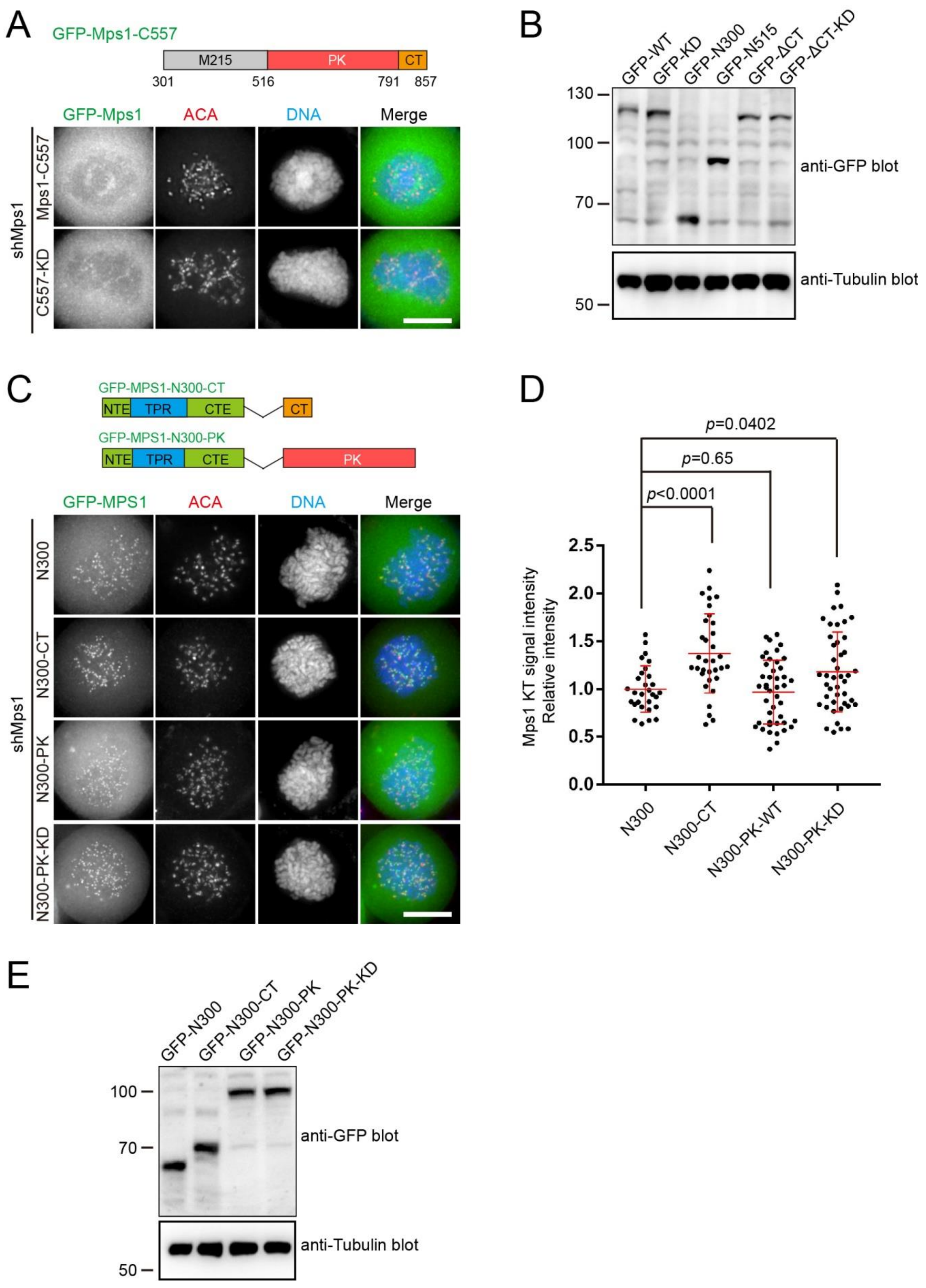
A
GFP-Mps1-C557
M215
PK
CT
301
516
791
857
GFP-Mps1
ACA
DNA
Merge
shMps1
Mps1-C557
C557-KD
B
GFP-WT
GFP-KD
GFP-N300
GFP-N515
GFP-ΔCT
GFP-ΔCT-KD
130
100
70
50
anti-GFP blot
anti-Tubulin blot
C
GFP-MPS1-N300-CT
NTE
TPR
CTE
CT
GFP-MPS1-N300-PK
NTE
TPR
CTE
PK
GFP-MPS1
ACA
DNA
Merge
shMps1
N300
N300-CT
N300-PK
N300-PK-KD
D
p=0.0402
p=0.65
p<0.0001
Mps1 KT signal intensity
Relative intensity
2.5
2.0
1.5
1.0
0.5
0.0
N300
N300-CT
N300-PK-WT
N300-PK-KD
E
GFP-N300
GFP-N300-CT
GFP-N300-PK
GFP-N300-PK-KD
100
70
50
anti-GFP blot
anti-Tubulin blot

Figure S3. Mps1 C-terminal fragment contributes to kinetochore localization.

(A) $Mps1^{C557}$ fragment does not have kinetochore localization activity. Representative immunofluorescence images of HeLa cells transfected with shMps1 and GFP-$Mps1^{C557\text{-}WT/KD}$ constructs as indicated. 36 hours post transfection, cells were treated with Nocodazole plus MG132 for 2 hours. Then cells were fixed and co-stained for ACA (red) and DNA (blue). Scale bar represents 10 μm.

(B and E) Western blot showing the comparable expression of different Mps1 constructs as indicated. 24 hours after transfection into HeLa cells, cell lysates were prepared. After separation by SDS-PAGE, samples were probed with the indicated antibodies.

(C) $Mps1^{CT}$ and $Mps1^{PK}$ fragments enhance kinetochore localization. Representative immunofluorescence images of HeLa cells transfected with shMps1 and different GFP-Mps1 constructs as indicated. 36 hours post transfection, cells were treated with Nocodazole plus MG132 for 2 hours. Then cells were fixed and co-stained for ACA (red) and DNA (blue). Scale bar represents 10 μm.

(D) Bar graph illustrating kinetochore intensity of different GFP-Mps1 fusion proteins treated as in C. Bars represent the mean kinetochore intensity (±SD) normalized to the values of Mps1-N300 (>30 cells from 3 independent experiments). Student's *t* test was used to calculate *p*-values.

Figure S4

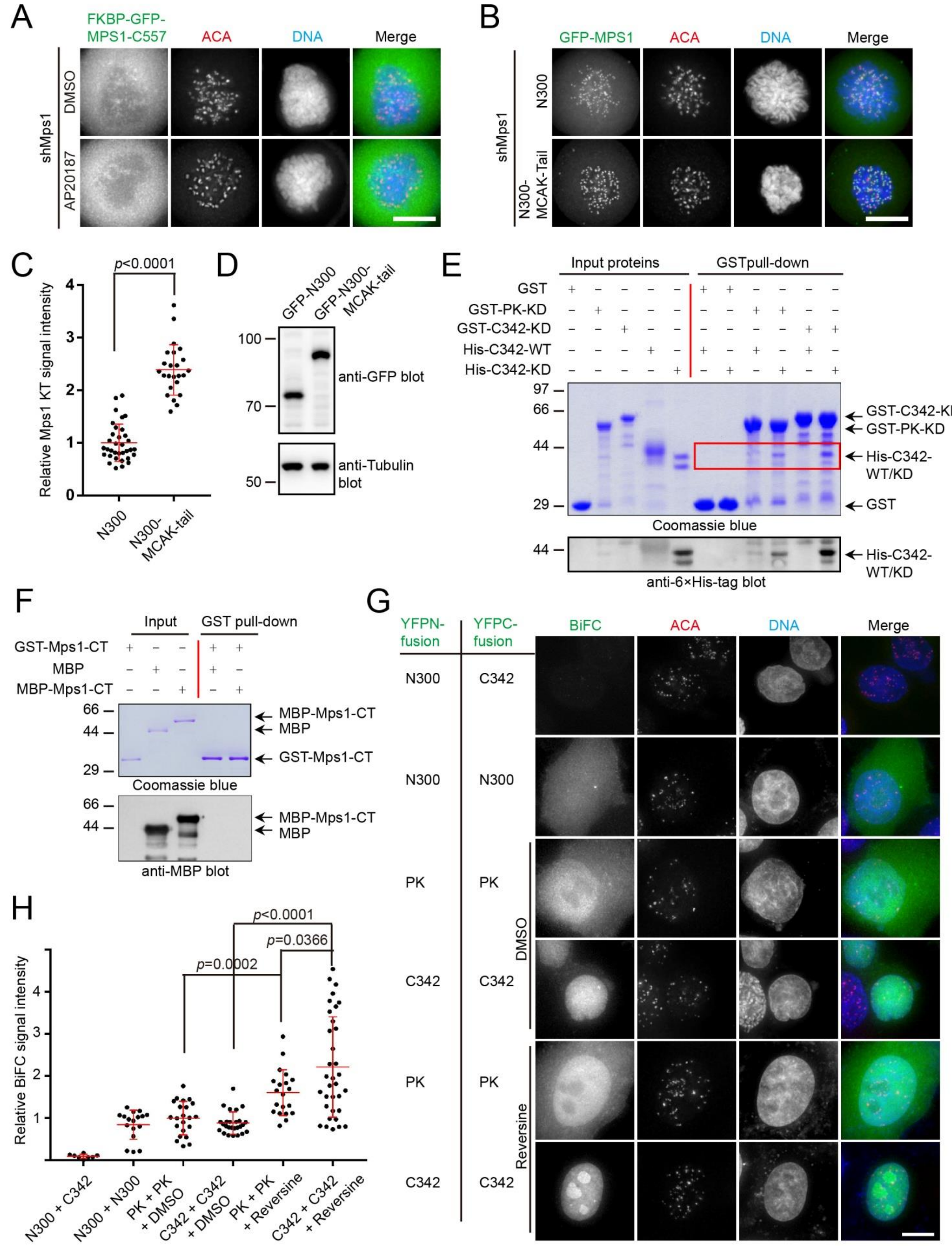

A
FKBP-GFP-MPS1-C557
ACA
DNA
Merge
shMps1
DMSO
AP20187
B
GFP-MPS1
ACA
DNA
Merge
shMps1
N300
N300-MCAK-Tail
C
p<0.0001
Relative Mps1 KT signal intensity
N300
N300-MCAK-tail
D
GFP-N300
GFP-N300-MCAK-tail
anti-GFP blot
anti-Tubulin blot
E
Input proteins
GSTpull-down
GST
GST-PK-KD
GST-C342-KD
His-C342-WT
His-C342-KD
GST-C342-KD
GST-PK-KD
His-C342-WT/KD
GST
Coomassie blue
anti-6×His-tag blot
F
Input
GST pull-down
GST-Mps1-CT
MBP
MBP-Mps1-CT
Coomassie blue
anti-MBP blot
G
YFPN-fusion
YFPC-fusion
BiFC
ACA
DNA
Merge
N300 C342
N300 N300
PK PK
C342 C342
DMSO
PK PK
C342 C342
Reversine
H
p<0.0001
p=0.0366
p=0.0002
Relative BiFC signal intensity
N300 + C342
N300 + N300
PK + PK + DMSO
C342 + C342 + DMSO
PK + PK + Reversine
C342 + C342 + Reversine

Figure S4. Mps1 C-terminal fragment contributes to Mps1 dimerization.

(A) Representative immunofluorescence images of HeLa cells transfected with shMps1 and shMps1 resistant FKBP-GFP-Mps1^{C557}. 36 hours post transfection, cells were treated with Nocodazole plus MG132 for 2 hours. One group of cells was treated with AP20187 for 30 minutes. Then cells were fixed and co-stained for ACA (red) and DNA (blue). Scale bar represents 10 μm.

(B) Representative images of HeLa cells transfected with shMps1 and shMps1 resistant GFP-Mps1^{N300} or GFP-Mps1$^{N300\text{-}MCAK\text{-}tail}$ construct as indicated. 36 hours post transfection, cells were treated with Nocodazole plus MG132 for 2 hours. Then cells were fixed and co-stained for ACA (red) and DNA (blue). Scale bar represents 10 μm.

(C) Bar graph illustrating kinetochore intensity of indicated fusion proteins treated as in B. Bars represent the mean kinetochore intensity (±SD) normalized to the values of GFP-Mps1^{N300} (>30 cells from 3 independent experiments). Student's *t* test was used to calculate *p*-values.

(D) Western blot showing the comparable expression of different Mps1 constructs as indicated. 24 hours after transfection into HeLa cells, cell lysates were prepared. After separation by SDS-PAGE, samples were probed with the indicated antibodies.

(E) GST-, GST-Mps1$^{PK\text{-}KD}$- or GST-Mps1$^{C342\text{-}KD}$-bound agarose beads were used as affinity matrices to absorb 6×His-tagged Mps1^{C342} truncation proteins purified from E. coli. Pull-downs were analyzed by SDS-PAGE and probed by anti-His-tag blotting.

(F) GST-Mps1^{CT}-bound agarose beads were used as affinity matrices to absorb MBP-tagged Mps1^{CT} truncation proteins purified from E. coli. Pull-downs were analyzed by SDS-PAGE and probed by anti-MBP-tag blotting.

(G) BiFC experiment indicates Mps1^{C342} fragment forms dimer. Representative immunofluorescence

images of HeLa cells co-expressing different YFPN- and YFPC-fusion constructs as indicated. 24 hours post transfection, cells were fixed and stained with ACA (red) and DAPI (blue). Scale bar represents 10 μm.

(H) Bar graph illustrating BiFC signal intensity of cells treated as in G. Bars represent the mean signal intensity (±SD) of cells analyzed (>30 cells from 3 independent experiments). Each dot represents one cell. Student's *t* test was used to calculate *p*-values.

Figure. S5

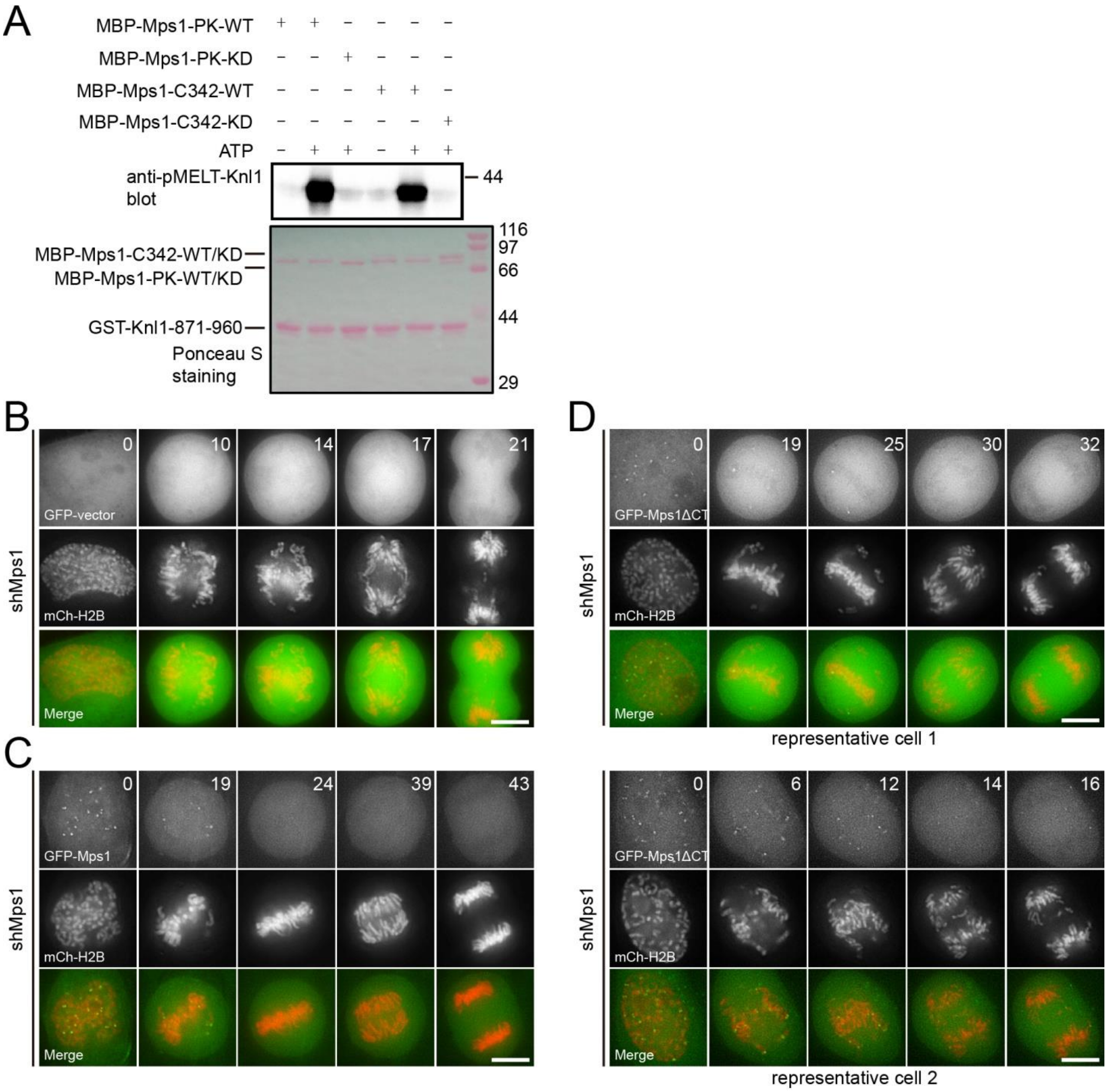
A
MBP-Mps1-PK-WT
MBP-Mps1-PK-KD
MBP-Mps1-C342-WT
MBP-Mps1-C342-KD
ATP
anti-pMELT-Knl1 blot
44
116
97
66
44
29
MBP-Mps1-C342-WT/KD
MBP-Mps1-PK-WT/KD
GST-Knl1-871-960
Ponceau S staining
B
shMps1
0
10
14
17
21
GFP-vector
mCh-H2B
Merge
C
shMps1
0
19
24
39
43
GFP-Mps1
mCh-H2B
Merge
D
shMps1
0
19
25
30
32
GFP-Mps1ΔCT
mCh-H2B
Merge
representative cell 1
0
6
12
14
16
GFP-Mps1ΔCT
mCh-H2B
Merge
representative cell 2

Figure S5. Mps1 C-tail is required for the functional integrity of SAC.

(A) *In vitro* phosphorylation of recombinant GST-Knl1$^{871\text{-}960}$ by different recombinant MBP-tagged Mps1 protein as indicated. After *in vitro* kinase reaction, the samples were analyzed by SDS-PAGE and Western blot. The upper panel shows the result of anti-pMELT-Knl1 blot and the lower panel shows Ponceau S staining of the transferred nitrocellulose membrane after SDS-PAGE electrophoresis.

(B, C and D) Representative stills illustrating mitotic progression in mCherry-H2B expressing cells depleted of endogenous Mps1 and rescued with GFP-vector (B), GFP-Mps1 (C) or GFP-Mps1$^{\Delta CT}$ (D) construct. Images were acquired at the indicated time points after the start of nuclear envelope breakdown. Scale bar represents 10 µm.
